# Genetic Adaptation of Schizothoracine Fish to the Phased Uplifting of the Qinghai–Tibetan Plateau

**DOI:** 10.1534/g3.116.038406

**Published:** 2017-02-14

**Authors:** Dongsheng Zhang, Mengchao Yu, Peng Hu, Sihua Peng, Yimeng Liu, Weiwen Li, Congcong Wang, Shunping He, Wanying Zhai, Qianghua Xu, Liangbiao Chen

**Affiliations:** * Key Laboratory of Aquaculture Resources and Utilization, Ministry of Education, College of Fisheries and Life Science, Shanghai Ocean University, China; † Key Laboratory of Sustainable Exploitation of Oceanic Fisheries Resources, Ministry of Education, College of Marine Sciences, Shanghai Ocean University, China; ‡ The Key Laboratory of Aquatic Biodiversity and Conservation of Chinese Academy of Sciences, Institute of Hydrobiology, Chinese Academy of Sciences, Wuhan, China; § Marine Biology and Biotechnology Laboratory, Qingdao National Laboratory for Marine Science and Technology, China

**Keywords:** Qinghai-Tibetan Plateau, adaptive evolution, hypoxia, Schizothoracine fish, GenPred, Shared Data Resources, Genomic Selection

## Abstract

Many species of Schizothoracine, a subfamily of Cyprinidae, are highly endemic to the Qinghai–Tibetan Plateau (QTP). To characterize the adaptive changes associated with the Schizothoracine expansion at high altitudes, we sequenced tissue transcriptomes of two highland and two subhighland Schizothoracines and analyzed gene evolution patterns by comparing with lowland cyprinids. Phylogenetic tree reconstruction and divergence time estimation indicated that the common ancestor of Schizothoracine fish lived ∼32.7 million years ago (MYA), coinciding with the timing of the first phase of QTP uplifting. Both high- and subhigh-Schizothoracines demonstrated elevated d*N*/d*S* ratios in the protein-coding genes compared to lowland cyprinids, from which some biological processes implicated in altitude adaptation were commonly identified. On the other hand, the highland and subhighland lineages presented drastically divergent landscapes of positively selected genes (PSGs), enriched with very different gene ontology (GO) profiles, including those in “sensory organ morphogenesis,” “regulation of protein ubiquitination,” “blood circulation,” and “blood vessel development.” These results indicated different selection pressures imposed on the highland and subhighland lineages of the Schizothoracine subfamily, with a higher number of genes in the high-altitude species involved in adaptations such as sensory perception, blood circulation, and protein metabolism. Our study indicated divergent genetic adaptations in the aquatic species facing the phased uplifting of QTP.

Being the largest and highest plateau on Earth, the QTP has undergone continuous uplifting during the India–Asia collision since ∼45 MYA (Li and Fang 1999; Favre *et al.* 2015). Dramatic climate and environmental changes ensued after the uplifts, including decreased oxygen pressure, lowered annual temperature, and increased ultraviolet radiation at the high altitudes. These factors imposed harsh physiological challenges to the endemic animals (Scheinfeldt and Tishkoff 2010). With recent advances in sequencing technologies and comparative genomics, a growing body of studies has been carried out to explore the genetic basis of environmental adaptation in the QTP organisms. These studies were initially focused on the native Tibetan people (Bigham *et al.* 2010, 2013; Simonson *et al.* 2010; Yi *et al.* 2010; Peng *et al.* 2011; Xu *et al.* 2011), and later on extended to various animal taxa endemic to the plateau, including yak (Qiu *et al.* 2012), frog (Yang *et al.* 2012), antelope (Ge *et al.* 2013), ground tit (Cai *et al.* 2013; Qu *et al.* 2013), pig (M. Li *et al.* 2013; Dong *et al.* 2014), Mastiff (Gou *et al.* 2014; Li *et al.* 2014), zokor (Shao *et al.* 2015), and lizard (Y. Yang *et al.* 2015). Various adaptive mechanisms have been revealed in these studies. For example, energy metabolism- (Bigham *et al.* 2010; Qiu *et al.* 2012; Ge *et al.* 2013; M. Li *et al.* 2013; Qu *et al.* 2013; Shao *et al.* 2015; Y. Yang *et al.* 2015) and blood vessel development-related genes (Dong *et al.* 2014; Gou *et al.* 2014; Shao *et al.* 2015) were found to undergo adaptive changes in multiple Tibetan species.

Fish are distributed in most parts of the Earth and often serve as models for adaptation studies. For example, cichlid fish provide excellent examples of diverse adaptive radiations (Henning and Meyer 2014) and Antarctic fish are taken as textbook examples for studying cold adaptation (Detrich *et al.* 2010). Species of Schizothoracine, a subfamily of Cyprinidae, dominate the rivers and lake drainages in QTP and its peripheral regions (Cao *et al.* 1981; He and Chen 2006). According to the degree of specialization of their morphological traits, the Schizothoracine fish are divided into three groups: primitive, specialized, and highly specialized, with the altitudes of their habitats increasing from the primitive to the highly specialized grade (Cao *et al.* 1981; He and Chen 2006; Qi *et al.* 2012). However, the “specialized” and “highly specialized” Schizothoracines are intermingled with each other in the high-altitude habitats. The specialized Schizothoracines are mainly distributed in the central part of QTP, whereas the primitive ones are found in the peripheral regions (Qi *et al.* 2012). Cao *et al.* (1981) suggested that the three phases of uplift of the Tibetan Plateau have contributed to the speciation of the Schizothoracine fishes, and that the degree of specialization of the Schizothoracines is closely associated with the environmental changes caused by the dramatic uplift of the Tibetan Plateau. Recent studies on the Tibetan fish confirmed that genetic adaptation to highland environments occurred in these fish. A comparative study showed that genes related to hypoxia and energy metabolism generally demonstrated rapid evolution, with many under positive selection in the specialized Schizothoracine fish *Gymnodiptychus pachycheilus* (L. Yang *et al.* 2015). Transcriptome studies on the Tibetan catfish and loach revealed similar results (Wang *et al.* 2015b; Ma *et al.* 2015). In terms of individual genes, genes that encoding hypoxia inducible factor 1α (Guan *et al.* 2014) and erythropoietin (Xu *et al.* 2016) are involved in hypoxic adaptation in high-altitude species. These studies indicated that, like the terrestrial animals, significant molecular modifications have taken place in the highland Schizothoracine fish to enable them to cope with hypoxia, ultraviolet light, and energy challenges in their aquatic environments.

Despite such recent progress, there are gaps in our knowledge regarding the evolutionary and adaptive aspects of Schizothoracine expansion in the QTP. For example, the divergence times between lineages of Schizothoracines have not been resolved with adequate molecular evidence, and current transcriptome studies are limited to single highland species with comparisons made to distantly related teleosts with available genomic information. Such comparisons have a limited power for investigating genetic adaptations due to substitution saturation and a lack of temporal resolution concerning the actions of natural selection. Instead, comparisons among closely related Schizothoracine species and other cyprinid species will allow for a stronger reconstruction of adaptive evolution linked to the QTP uplift.

As genome sequencing data are not available for the Schizothoracine fishes, transcriptome sequencing is an effective option for genome-wide comparative studies of highland adaptation. In this study, we sequenced transcriptomes of multiple tissues from four Schizothoracine fishes, including two high-altitude and specialized ones (*Gymnocypris dobula* and *Ptychobarbus kaznakovi*, termed as highland species), and two subhigh-altitude and primitive ones (*Schizothorax gongshanensi* and *S. prenanti*, termed as subhighland species). Unigenes from the Schizothoracines were compared to the orthologous genes from two lowland, non-Schizothoracine fish, zebrafish (*Danio rerio*) and grass carp (*Ctenopharyngodon idella*). We estimated the divergence times among the tested species and revealed differential evolutionary changes in the genes of the highland and subhighland lineages, which coincided with the phased uplifting of the QTP.

## Materials and Methods

### Sample collection and library preparation

The location in longitude and latitude coordinates for the sampling site for the four Schizothoracine fishes, *G. dobula*, *P. kaznakovi*, *S. gongshanensi*, and *S. prenanti* was documented using a handheld GPS (Explorist 210, Magellan Corp.); the geographic information for the sampling sites is in Table 1. Fishes of the Schizothoracine were collected in August 2010, and the oxygen concentration of the sampling site was measured. For each species, 2–15 individuals were captured from the same sampling location [data were shown in our previous study (Xu *et al.* 2016)], and fish of similar size were selected for tissue dissection. Four tissues of different developmental ontogenies (brain, liver, muscle, and spleen) were sampled from each fish to maximize the number of expressed protein genes of the species. Tissues of the same individual from each species were used for the analysis. These tissues were quickly biopsied and placed in RNAlater (QIAGEN), and were stored at −80° on arrival at the laboratory till RNA extraction. Experimental protocols involving live animals were approved by the Ethics Committee for the Use of Animal Subjects of Shanghai Ocean University.

**Table 1:** Sampling locations for the Schizothoracines

Species	Location	Geographic Coordinates	Altitude (m)	Group
*G. dobula*	Lake Duoqing, Tibet	28°03.37′, 89°17.83′	4506 ± 9	Highland
*P. kaznakovi*	Xialaxiu, Qinghai	32°38.13′, 96°33.8′	3911 ± 9	Highland
*S. gongshanensis*	Gongshan, Yunnan	27°39.23′, 98°43.12′	1212 ± 9	Subhighland
*S. prenanti*	Ya’an, Sichuan	29°98.48′, 103°01.19′	950 ± 3	Subhighland

Total RNAs from tissues were extracted using TRIzol reagent (Invitrogen Corp., Carlsbad, CA). The quality of RNA was assessed by measuring the RNA Integrity Number (RIN) using a Bioanalyzer Chip RNA 7500 series II (Agilent). Samples with RIN > 8.0 were used for sequencing. The concentration of total RNA was determined with a Qubit fluorometer (Life Technologies). Three micrograms of total RNA from each sample was applied to isolate the polyA+ RNA and the mRNA-Seq library was constructed using the TruSeq RNA Sample Prep Kit (Illumina). The libraries were quantified using a 2100 bioanalyzer (Agilent Technologies). Similar quantities from each library were sequenced using the Illumina Hiseq1500 platform following the manufacturer’s instructions.

### Sequence assembly and annotation

Clean read pairs were obtained by removing adaptors and low-quality reads (quality scores < 20) using Trimmomatic (Bolger *et al.* 2014), and contigs were generated by pooling all transcriptomes of four tissues for each species using the Trinity package with default settings (Grabherr *et al.* 2011). All sequence reads have been deposited in the National Center for Biotechnology Information (NCBI) Sequence Read Archive database under the accession number SRP095660. Partial and complete open reading frames (ORFs) were predicted using the transdecoder script from the Trinity package with a criterion of minimum length of 50 amino acids.

Protein and coding DNA sequences (CDS) of *D. rerio* and *C. idella* were downloaded from the following websites: http://www.ensembl.org/Danio_rerio and http://www.ncgr.ac.cn/grasscarp/, respectively. The longest transcript was chosen for the downstream analysis if multiple transcripts were present in a gene. To produce reliable annotations for the contigs, BLASTP bidirectional best hit (BBH) (Camacho *et al.* 2009) was applied between each deduced protein sequence dataset of Schizothoracine fish and the reference protein dataset of *D. rerio* (Genome Reference Consortium Zebrafish Build 10 from Ensembl database) with *e*-value cutoff of 10^−5^. Protein sequences without annotations were not included in further analysis.

### Identification of the orthologous genes and sequence alignment

The protein sequences from the six species were processed by OrthoMCL (Li *et al.* 2003) to identify the orthologous gene families. The resulting orthologous gene families were aligned at the protein level using the Prank program (Loytynoja and Goldman 2008). The corresponding codon alignments were produced using PAL2NAL (Suyama *et al.* 2006) and the least reliably aligned regions were removed using the Gblocks program (Castresana 2000). The trimmed alignments with lengths >150 bp were kept for further analysis.

### Phylogenetic tree construction and divergence time estimation

To establish phylogenetic relationships of the Schizothoracine fish and the cyprinids, we used the single-copy orthologous genes shared by all six species to reconstruct the phylogenetic tree. After multiple sequence alignment and trimming, the aligned CDS sequences of the single-copy orthologs were concatenated into a “supergene” for each species. Fourfold degenerate sites from the supergenes were used for phylogenetic tree reconstruction and divergence time estimation. Fourfold degenerate sites are the third sites across the eight fourfold degenerate codon families, including glycine-GCN, leucine-CTN, valine-GTN, arginine-CGN, threonine-ACN, alanine-GCN, serine-TCN, and proline-CCN, for which any nucleotide substitution is synonymous. The fourfold degenerate sites of the conserved amino acids of the aligned single-copy orthologous genes were extracted and concatenated using in-house Python scripts. We used Modeltest (Posada and Buckley 2004) to select the best substitution model (GTR + γ + I). Maximum likelihood method was applied for phylogenetic tree reconstruction using PAUP (Swofford 2003). The tree topology was assessed by 1000 bootstrap replicates.

The MCMCtree program implemented in the PAML v4.8 package was used to estimate the divergence times among the species using a maximum likelihood method (Yang 2007). The substitution rate was estimated using the BASEML program in PAML. The overall substitution rate (rgene γ) and rate-drift parameter (sigma2 γ), were set as: G (1, 8.7) and G (1, 0.638), respectively, and estimated using the strict molecular-clock assumption with a root age of 63.8 Ma, which was referenced from a previous study (Betancur *et al.* 2015). The independent rates model (clock = 2), which assumes uncorrelated relaxed molecular clock, was used to specify the prior of rates among the internal nodes, which followed a log-normal distribution. The three parameters (birth rate λ, death rate µ, and sampling fraction ρ) in the birth–death process with species sampling were specified as 1, 1, and 0, respectively. A loose maximum bound for the root was set between 48.6 and 94.9 MYA. The former was the record of the oldest Cyprinidae fossil (Patterson 1993); the latter was estimated to be the date of the most recent common ancestor for Cypriniformes (Near *et al.* 2012) and was taken as the upper limit for divergence time of Cyprinidae in a recent study (Zhao *et al.* 2016). We also incorporated the estimated divergence time between zebrafish and grass carp (49–54 MYA) (Wang *et al.* 2015a) as a reference time to calibrate the divergence times of other nodes. The first 50,000 cycles in MCMCTREE were discarded as burn-in, and every 50 cycles were sampled to obtain a total of 10,000 samples.

### Calculation of lineage-specific dN/dS values, and identification of GO categories with elevated dN/dS

Adaptive evolution could be indicated by an increased ratio of nonsynonymous to synonymous substitutions (d*N*/d*S*), especially for the genes that related to environmental adaptation. To estimate lineage-specific d*N*/d*S* values, the codeml program in the PAML package (Yang 2007) with the free-ratio model (M1) was run on codon alignment of each single-copy orthologous gene and the concatenated codon alignments of all these genes according to the reconstructed tree topology. The resulting codeml data, including the *N*, *S*, d*N*, d*S*, and d*N*/d*S* values for the genes of each lineage, were retrieved for further analysis. Genes with *N* * d*N* or *S* * d*S* < 1 or d*S* > 1 were filtered following the approach used in a previous study (Goodman *et al.* 2009). Comparisons of the d*N*/d*S* values among the lineages were conducted using the Wilcoxon rank-sum test.

We further characterized selection constraints on GO categories in Schizothoracine fishes by calculating the average d*N*/d*S* values for GO categories. We assigned GO annotation to the orthologs according to zebrafish genome annotation, which was downloaded from ENSEMBL website (http://www.ensembl.org). GO categories containing at least 30 single-copy orthologous genes were included in further analysis. For each GO category, codon alignments of all orthologs belonging to this GO category were concatenated into a “supergene” and the average d*N*/d*S* values for this GO category were estimated by running a free-ratio model on the “supergene.” The GO categories with relatively increased or decelerated d*N*/d*S* values were identified by binomial tests, as previously described by the Chimpanzee Sequencing and Analysis Consortium (2005).

### Positive selection analysis

Since the branch-site model in the codeml program (Zhang *et al.* 2005) has the advantage of detecting positive selection that affects only a few sites on a prespecified (foreground) branch of the species tree, this model was used to detect positive selection in the single-copy orthologous genes. To detect PSGs in highland Schizothoracines, the highland species were designated as the foreground lineages and compared to lowland lineages for the branch-site model analysis. The tree file for the branch-site model is [(*G. dobula* #1, *P. kaznakovi* #1), *C. idella*, *D. rerio*] with the foreground species labeled with #1. In a similar way, we compared subhighland Schizothoracines with lowland species to detect potential genes under positive selection in subhighland species with tree file [(*S. gongshanensis* #1, *S. prenati* #1), *C. idella*, *D. rerio*]. The likelihood rate test (LRT) was applied to detect positive selection on the foreground branches. PSGs were inferred only if their *P*-values were <0.05 after FDR normalization using the Benjamini–Hochberg approach (Benjamini and Hochberg 1995). GO enrichment analyses of the PSGs were performed using a hypergeometric method (Rivals *et al.* 2007).

### Protein–protein interaction (PPI) network

The PSGs of interest were mapped to the human genome. The PPI information for humans recorded in the InnateDB (Breuer *et al.* 2013) was applied to obtain the network topology using Cytoscape3.3.0 (Killcoyne *et al.* 2009).

### Data availability

The raw RNA-Seq reads were deposited at NCBI Sequence Read Archive under the accession SRP095660. 

## Results

### Sequencing, de novo assembly, and annotation of the transcriptomes

Sequencing of the four Schizothoracine fishes, *G. dobula*, *P. kaznakovi*, *S. gongshanensi*, and *S. prenanti*, generated 106,198,802–255,610,690 reads using the Illumina Hiseq1500 platform. Detailed information regarding the sequencing and assembly of the transcriptomes is summarized in Table 2. The assembled contigs were annotated according to the zebrafish reference protein dataset using the BBH method. There were 13,411 and 16,322 contigs annotated for *P. kaznakovi* and *G. dobula*, respectively, with 15,501 and 17,063 contigs for *S. gongshanensi* and *S. prenanti*, respectively. The large number of annotated genes obtained by the transcriptome sequencing for each species indicated a significant portion of the protein-coding genes of these species were obtained.

**Table 2:** Summary of the transcriptome sequencing data

Species	*P. kaznakovi*	*G. dobula*	*S. gongshanensis*	*S. prenati*
Raw reads	106,198,802	213,299,257	255,610,690	140,646,372
Clean reads	58,285,155	125,564,625	134,727,852	69,059,574
No. of contigs	117,614	151,970	165,653	115,164
Assembly size (Mb)	90.7	139.6	164.8	114.8
Mean length (bp)	771	918	995	997
Median length (bp)	448	452	498	469
>300 bp contigs	81,745	103,510	117,336	80,277
>500 bp contigs	53,952	71,099	82,663	55,183
>1 kb contigs	28,885	43,431	52,364	35,040
N50 (bp)	1266	1736	1865	1950
N90 (bp)	305	327	358	350

No., number.

### The phylogenetic relationship and divergence time between the highland and subhighland Schizothoracines

**Figure 1:**
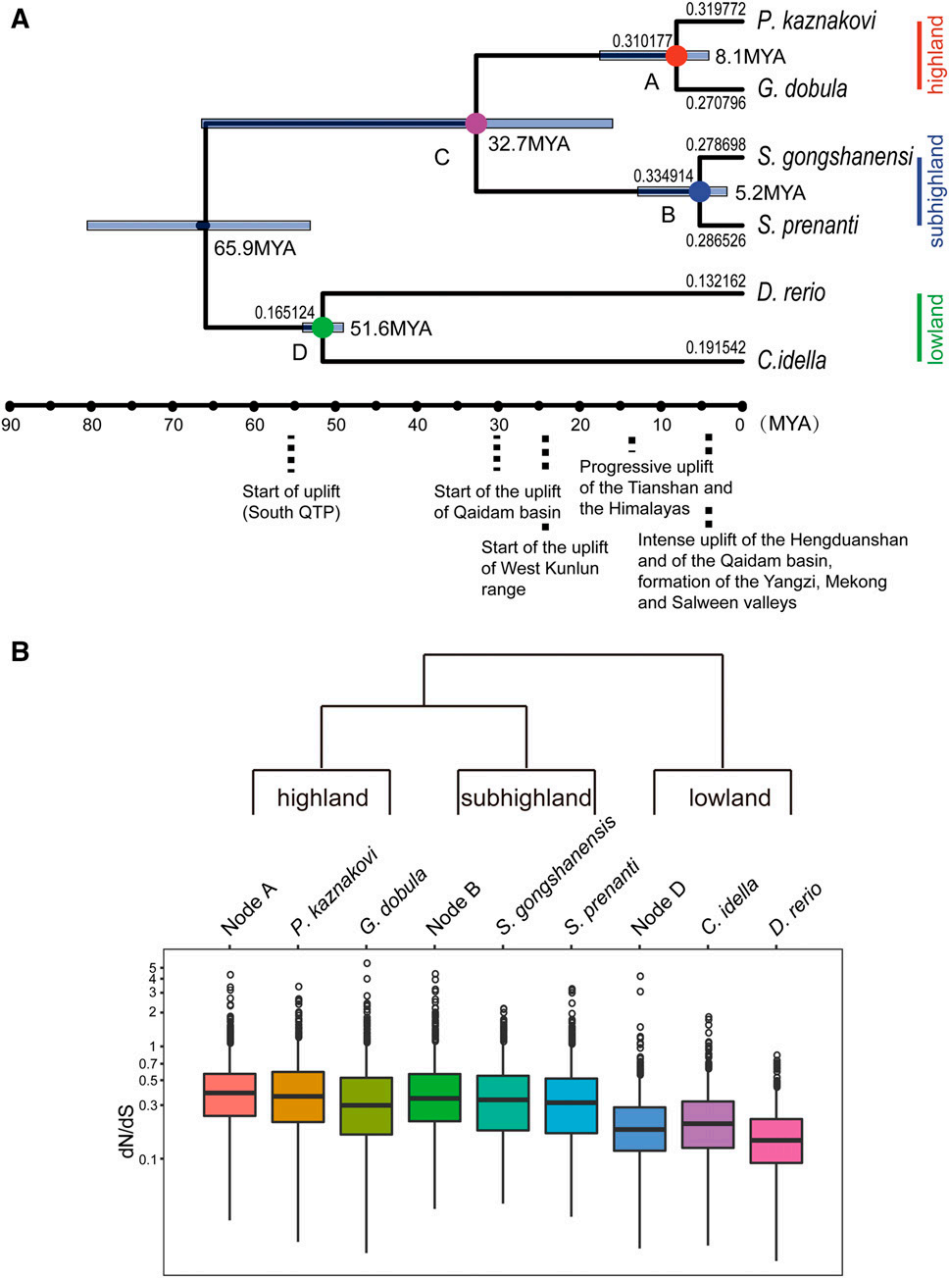
(A) Phylogenetic tree with divergence times and d*N*/d*S* ratios for each node. Phylogenetic tree was derived from fourfold degenerate sites of concatenated sequences from 7532 single-copy orthologs by the maximum likelihood method. Divergence times were estimated using MCMCtree, and d*N*/d*S* ratios were estimated from the concatenated codon alignment deduced from all single-copy orthologs using a free-ratio model in the codeml program. Geographic events are denoted according to Favre *et al.* (2015) and Li and Fang (1999). (B) The distributions of d*N*/d*S* ratios of the single-copy orthologs for each node. d*N*/d*S* ratios for all branches were estimated from each single-copy ortholog using the free-ratio model in codeml.

A total of 16,766 orthologous gene families were clustered using the ORTHOMCL software, of which 8049 were single-copy orthologous among the six species. After multiple sequence alignment and trimming of low quality alignment, 7532 single-copy orthologous genes were retained for further analysis. They were concatenated into a single supergene and 753,740 fourfold degenerate sites were used for phylogenetic tree reconstruction and divergence time estimation (Figure 1A). All nodes were supported with bootstrap values of 100, indicating a well-resolved relationship between the six species (Figure 1A). It is evident that the two specialized species, *G. dobula* and *P. kaznakovi*, formed one lineage (highland lineage) and that the two primitive species, *S. gongshanensis* and *S. prenanti*, formed another lineage (subhighland lineage), with the non-Schizothoracine cyprinids diverging earlier.

MCMCtree software was applied to estimate the divergence time for each node using the fourfold degenerate sites based on the well-supported phylogenetic tree we reconstructed. The divergence time was dated ∼8.1 MYA (95% C.I. of 4.1–17.5 MYA) for *P. kaznakovi* and *G. dobula*, ∼5.2 MYA (95% C.I. of 1.8–12.8 MYA) for *S. gongshanensis* and *S. prenanti*, and the common ancestor for the four Schizothoracine fish was dated ∼32.7 MYA (95% C.I. of 15.9–66.4 MYA) (Figure 1A).

### Elevated dN/dS ratios in the lineages of Schizothoracine fishes

The ratio of nonsynonymous to synonymous substitutions (d*N*/d*S*) for each ortholog was evaluated for all branches of the reconstructed phylogenetic tree using the free ratio model in PAML (Yang 2007). The Schizothoracine fish and their most recent ancestors (node A and node B) exhibited significantly higher d*N*/d*S* values than the lowland cyprinid species (Wilcoxon rank-sum tests, *P*-values < 2.2e−16) (Figure 1B). Furthermore, we calculated the d*N*/d*S* ratios for each branch using a concatenated codon alignment of 7532 single-copy orthologs, which also indicated that the Schizothoracine fish lineages had higher d*N*/d*S* ratios than the lowland cyprinid lineages (Figure 1A).

**Figure 2:**
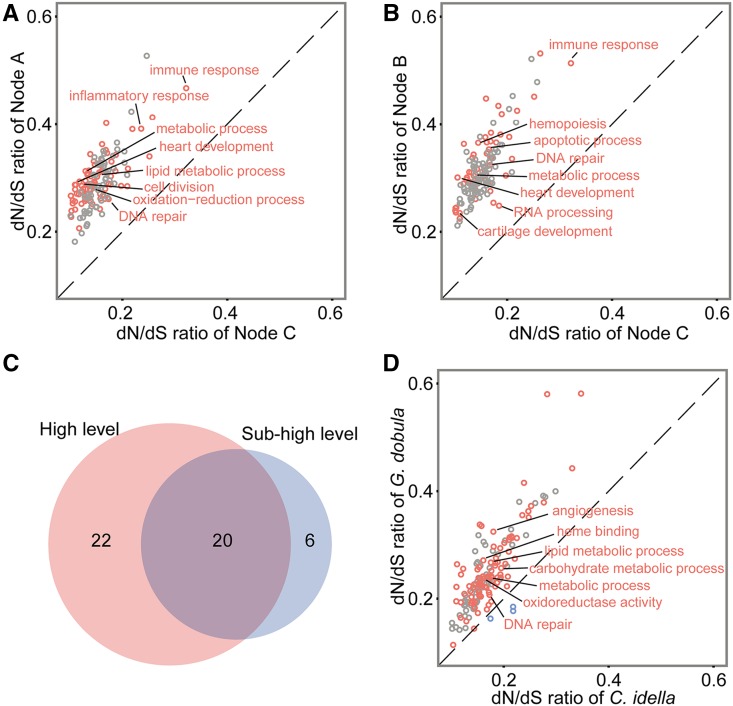
Comparisons of the d*N*/d*S* ratios between nodes based on GO categories. GO categories with significantly higher mean d*N*/d*S* ratios on the *x*-axis and *y*-axis are represented by blue and red dots, respectively. (A) Node A *vs.* node C; (B) node B *vs.* node C; (C) Venn diagram showing the overlapping of GO biological processes with elevated d*N*/d*S* ratios in the highland and subhighland lineages; (D) *G. dobula vs. C. idella*. GO, gene ontology.

We then classified the orthologous genes by assigned GO terms and estimated average d*N*/d*S* values for 150 GO categories that contained >30 single-copy orthologous genes. We screened for those GO categories that underwent rapid evolution in the most recent ancestors of the current Schizothoracine fish (Node A and Node B) by comparing with their common ancestor (Node C). We detected 42 and 26 GO categories of Biological Process (BP), which showed significantly increased d*N*/d*S* ratios (*P* < 0.05, binomial test) in the Node A/Node C and Node B/Node C comparisons, respectively (Figure 2, A and B). Among them, 20 GO categories overlapped (Figure 2C and Supplemental Material, Table S1). The common GO categories included “immune response,” “ion transport,” “metabolic processes,” “heart development,” and “DNA repair,” indicating that genes in these categories may be under higher evolutionary pressure than their most recent ancestors during the QTP uplift. There were 22 GO categories, including “lipid metabolic process,” “oxidation-reduction process,” and “protein phosphorylation,” that were found to have elevated d*N*/d*S* ratios in the highland species, many of which are known to be involved in homeostasis regulation when fish face low temperature stress (Chen *et al.* 2008; Hu *et al.* 2015). On the other hand, six GO categories, such as “regulation of apoptotic process,” “hemopoiesis,” and “cartilage development” appeared to have increased d*N*/d*S* ratios in the subhighland species. Furthermore, we compared d*N*/d*S* ratios of the terminal branches of highland Schizothoracine species to subhighland Schizothoracine species. As shown in Figure S1, there were 61 *vs.* 35 GO categories with elevated d*N*/d*S* ratios in *G. dobula* and *S. prenanti*, respectively. When *G. dobula* was compared to a lowland non-Schizothoracine species, *C. idella*, *G. dobula* apparently had drastically more GO categories with elevated d*N*/d*S* ratios than *C. idella* (86 in *G. dobula vs.* only 4 in *C. idella*) (Figure 2D).

### Functional divergence of the positive selection genes in the Schizothoracines of high and subhigh altitudes

**Figure 3:**
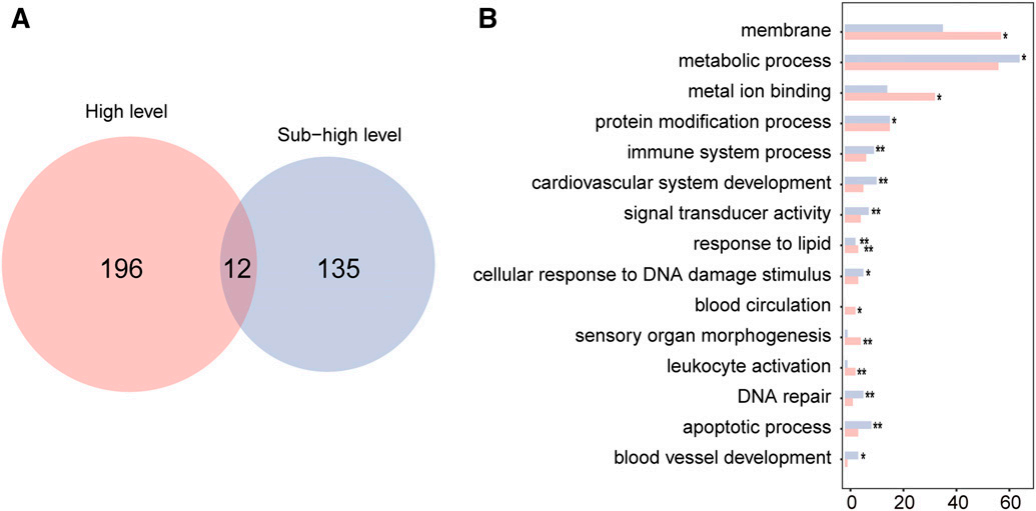
Differentially enriched GO profiles in PSGs of the highland and subhighland lineages. (A) Venn diagram showing distinct PSG sets identified from the highland and subhighland Schizothoracines. (B) Distribution of GO classification of PSGs in the two Schizothoracine lineages. **P-value < 0.01, *P-value < 0.1. GO, gene ontology; PSG, positively selected genes.

Elevated d*N*/d*S* values might be due to relaxation in purifying selection or intensification of positive selection, or some combination of both. As positive selection is crucial in adapting to a changing environment, we further investigated genes under positive selection in the Schizothoracine fish. We carried out branch-site model likelihood ratio tests on 7532 single-copy orthologous gene sets by setting either highland Schizothoracine species or subhighland Schizothoracine species as the foreground against the two lowland non-Schizothoracine species. We identified 208 and 147 PSGs from the highland and subhighland branches, respectively. The two PSG sets overlapped by only 12 genes (Figure 3A and Table S2), including immunoglobulin heavy constant δ (*ighd*) and iroquois homeobox 2a (*irx2a*); the former is involved in the immune response and the latter in eye development. Only three PSGs in our study were found to overlap with a previous study on a Schizothoracine fish, *Gy*. (L. Yang *et al.* 2015), probably because we used more closely related species for comparison. Nevertheless, genes related to hypoxia and energy metabolism were populated in both theirs and our study, indicating that these Schizothoracine fishes faced similar evolutionary pressures.

We classified the PSGs by GO categories and plotted the distribution of the PSGs among the GO categories that are comprised of at least three PSGs. In both PSG sets, GO categories “metabolic processes,” “membrane,” “ion binding,” “protein modification process,” “immune system process,” and “cardiovascular system development” were among the ones that contained the largest numbers of PSGs (Table S3 and Table S4), coinciding with the findings that genes in these GO categories or closely related GO categories had high d*N*/d*S* ratios in both the highland and subhighland species.

We then performed GO enrichment tests on the two PSG sets. We identified GO categories that were specifically enriched in the highland species (Figure 3B), such as “membrane,” “sensory organ morphogenesis,” and “blood circulation,” and several biological processes were found to be enriched in the subhighland PSGs (Figure 3B), among which were “blood vessel development,” “cardiovascular system development,” and “metabolic process.” Very few GO categories, including “respond to lipid,” were found to be significantly enriched in both. The disparate distribution of biological processes of the PSGs suggested divergent adaptation strategies deployed by the highland and subhighland Schizothoracines in evolution.

### PSGs related to cardiovascular development and hypoxic response in the highland and subhighland species

The heavy coverage of developmentally related biological processes in the enriched GO terms in the *G. dobula* and *P. kaznakovi* lineage (Figure 3B) suggested that changes in developmental programs played important roles in high-altitude adaptation. We found seven and twelve PSGs involved in various aspects of “cardiovascular system development” in the highland and subhighland species, respectively, with no genes in common in the two sets (Table S3 and Table S4). Furthermore, PSGs involved in “blood circulation” were restricted to highland species, including *cmlc1*, *emp2*, *enpep*, and *sgol1*. Five PSGs annotated to “blood vessel development” were present in the subhighland species, including *sars*, *nkx2.5*, *mtdha*, *dab2*, and *lpar6b*, while only one gene (*hdac6*) was identified in the highland species. The divergence in PSG distribution was also present in “leukocyte activation,” “apoptotic process,” and other GO categories. The different PSGs related to these biological processes implied differential adaptations in blood and cardiovascular developmental processes in the high and subhigh Schizothoracines.

Another noticeable difference between the highland and subhighland species is the PSGs involved in hypoxia responses. Adaptation to hypoxic conditions apparently constituted a large portion of adaptation events occurring to the highland species. We compared PSGs identified in our study with a hypoxia response gene list (containing a total of 3206 genes) developed from a study on the gray wolf (Zhang *et al.* 2014) and the HypoxiaDB (Khurana *et al.* 2013). In the highland and subhighland species, there were 28 and 21 PSGs found to be hypoxia responsive, respectively, and the two sets overlapped by zero genes (Table S5). Again, the results indicated diverged evolution in the hypoxia-related genes in the two Schizothoracine lineages.

**Figure 4:**
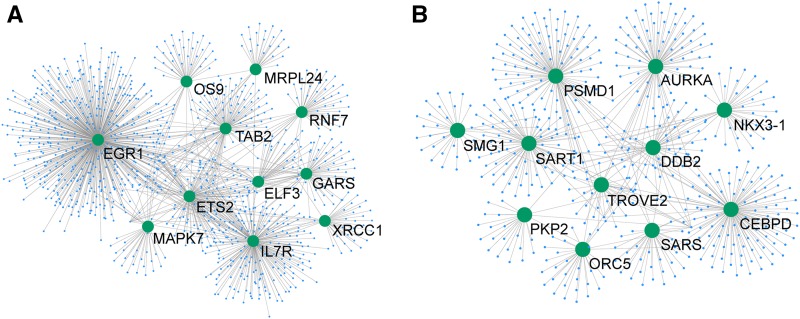
Protein–protein interaction network showing PSGs (indicated by green circular dots) that are related to hypoxic response. Only genes with >20 interacting relations within the network are shown. The protein–protein interaction information was extracted from InnateDB and the network topology was drawn by Cytoscape3.3.0 (Killcoyne *et al.* 2009). (A) The highland lineages; (B) the subhighland lineages. PSGs, positively selected genes.

To gain a view of interaction network between the hypoxia-related PSGs, we retrieved the PPI information from innateDB (Breuer *et al.* 2013) and constructed an interacting network for the hypoxic response genes from both the highland and the subhighland Schizothoracines. Individual PSGs varied widely in the number of proteins they interacted with. The PSGs with >20° of interaction are shown in Figure 4. *egr1*, which plays important roles in ischemia and hypoxia (Yan *et al.* 2000), has the most degrees (518°) in highland species. For both networks, nine PSGs are involved in various metabolic processes, including tRNA synthetases (*sars* and *gars*), mitogen-activated protein kinase (*mapk7*), and the proteasome (*psmd1*). This suggests important roles for metabolism in hypoxic responses in the Schizothoracines driven by the uplifting of the QTP. Transcriptional regulators involved in DNA repair were also found in both networks, such as *xrcc1* (Saadat *et al.* 2008) in the highland species and *ddb2* (Inoki *et al.* 2004) in the subhighland species.

## Discussion

Through evolutionary analysis of >11,000 unique contigs expressed in the four Schizothoracines from habitats at different altitudes, we gained genetic insights into the adaptation of these fishes at high altitudes.

We investigated the phylogenetic relationship between the selected Schizothoracines with closely related cypriniform fish. The four Schizothoracine fish are grouped by the grade of specialization (Cao *et al.* 1981), with the specialized (*P. kaznakovi*) and the highly specialized (*G. dobula*) sharing a recent common ancestor, and the two primitive species grouped together. Divergence times among these groups were previously estimated based on mitochondrion-coded genes and the results are controversial, probably due to variable and elevated mutation rates and the small size of mitogenomes when compared to genomic data. For example, the common ancestor of the schizothroacine subfamily was estimated to live at the end of the Late Cretaceous (68.2 MYA) by Y. Li *et al.* (2013), or at the Oligocene-Miocene boundary (∼23 MYA) or older by Ruber *et al.* (2007) and Wang *et al.* (2016). As the first attempt to characterize the phylogenetic relationships and divergence times of Schizothoracines on the basis of transcriptomic data, our results indicated that the common ancestor of the four Schizothoracines lived at the early part of the Oligocene (32.7 MYA), when the QTP was in the first phase of uplift (Favre *et al.* 2015; Li and Fang 1999). The common ancestor of the two primitive Schizothoracines, *S. prenanti* and *S. gongshanensis* (which belong to the genus *Schizothorax*), was estimated to be ∼5.2 MYA, which has been previously estimated to be in the Late Miocene (∼10 MYA) (He *et al.* 2004; Wang *et al.* 2016). Our results suggested that the common ancestor of *P. kaznakovi* and *G. dobula* diverged ∼8.1 MYA, when the QTP was in the second phase of uplift. The descendants of this lineage then experienced continued habitat uplifting in the QTP and presumably experienced more extensive selective pressure. We need to point it out that, although this analysis encompassed the largest number of genes from the Schizothoracines analyzed to date, the divergence time estimation is still inconclusive in our study. For example, the C.I. of divergence time for the common ancestor of Schizothoracines ranged from 15.9 to 66.4 MYA. Several factors may affect the estimation accuracy, for example, the actual date of the fossils used in the estimation, inconsistency of the reference times, or evolutionary rate variation in different branches (Seo *et al.* 2002; Bedford and Hartl 2008). Accurate fossil records and sequence information from more Schizothoracines and other closely related cyprinid species will be helpful in the estimation of divergence times for the Schizothoracines.

In this study, using the four Schizothoracines of different altitudes, we found that the d*N*/d*S* ratios in these Schizothoracines are commonly higher (d*N*/d*S* ≈ 0.3) than the non-Schizothoracine cyprinids, *D. rerio* and *C. idella* (d*N*/d*S* ≈ 0.16), indicating a general trend of increase in the evolutionary pressure in the Schizothoracines since the mid-Eocene. The overall elevation of d*N*/d*S* ratios might be related to the environmental changes associated with the first phase of QTP uplift. Using the GO enrichment analysis, we revealed the biological processes that have elevated d*N*/d*S* ratios in both the highland and subhighland lineages. We found that ∼50% (20/42) and 80% (20/26) of biological processes with elevated d*N*/d*S* ratios in the highland and subhighland lineages are in common, respectively (Figure 2C). The common processes are involved in the immune response, metabolic processes, DNA repair, and cardiovascular development. The results suggest that both the primitive and specialized Schizothoracines likely experienced certain types of common selection pressure during their evolution. Metabolic processes, DNA repair, and cardiovascular development have been proposed to be involved in high-altitude adaptation in other studies (Bigham *et al.* 2010; Qiu *et al.* 2012; Ge *et al.* 2013; M. Li *et al.* 2013; Qu *et al.* 2013; Shao *et al.* 2015; Y. Yang *et al.* 2015). This phenomenon may be partially attributable to the fact that the common ancestor of the four species lived at the time when the QTP was in the first phase of elevation, as illustrated in Figure 1A.

Analyses of the PSGs identified from the highland and subhighland lineages yielded significantly different landscapes of biological processes that were potentially under positive selection, as inferred by the little overlapping of the PSGs and the disparate distribution of the enriched biological processes (Figure 3B). These results suggested divergent evolutionary processes in highland and subhighland Schizothoracines. Previous phylogenetic studies indicated that the morphologically primitive Schizothoracine groups might have had independent origins and migrated independently of the morphologically specialized and highly specialized groups (Yonezawa *et al.* 2014; Wang *et al.* 2016). The divergent adaptive evolution revealed by this study supported these findings.

Highland environments are characterized by low oxygen concentration, cold, and ultraviolet radiation. Tolerance to hypoxia and cold may be linked to both the pathways of oxygen supply and demand. Oxygen supply is directly related to cardiovascular and red blood cell development, while oxygen demand is mainly linked to metabolic processes. Recent molecular studies on *epo* (Xu *et al.* 2016) and *hif1ab* (Guan *et al.* 2014) in the Schizothoracine species indicated that blood and cardiovascular system development play important roles in highland adaptation in fish. We did not find the gene *hif1ab* to be statistically significant as a positive selection gene in this study, which may be a result of the different species used in the PAML analysis between the two studies (Guan *et al.* 2014). Xu *et al.* (2016) reported that genes for erythropoietin and erythropoietin receptor in *G. dobula* are under weak selection, with *P*-values of LRT slightly over 0.05. Thus, neither gene was identified in current study due to the strict statistical measures enforced. We did find that hemoglobin ζ (*hbz*), which is involved in oxygen transportation, has signatures of positive selection in highland Schizothoracine species, indicating that adaptation in oxygen-binding efficiency might have occurred in the highland species. On the other hand, nine hypoxic responsive PSGs with the highest degrees of PPIs in Figure 4 were found to also be related to metabolic processes in the highland Schizothoracines, indicating that metabolic network and hypoxic responses are interconnected processes for adapting to the highland environments of the QTP. Further studies focusing on functional characterization of the PSGs will help to identify the key factors contributing to highland adaptation in the fishes.

In summary, we found that highland and subhighland lineages of the Schizothoracine subfamily experienced divergent adaptation during their evolution in the QTP region, and that genes related to highland adaptation, including metabolic processes, cardiovascular system development, and DNA repair, were under rapid evolution and/or have signs of positive selection. However, this study dealt only with sequence variations among the species. Gene expression changes also play important roles in highland adaptation (L. Yang *et al.* 2015; Shao *et al.* 2015), and this was not addressed in this study due to the lack of species or individuals from well-controlled environments. Studies on more species from similar environments and altitudes would help to identify complete mechanisms of highland adaptation in these fish.

## Supplementary Material

Supplemental material is available online at www.g3journal.org/lookup/suppl/doi:10.1534/g3.116.038406/-/DC1.
