# Supplementary figures and images for: Genetic Adaptation of Schizothoracine Fish to the Phased Uplifting of the Qinghai–Tibetan Plateau
Source: G3 (Bethesda). 2017 Feb 14;7(4):1267–76. doi: 10.1534/g3.116.038406 (PMC5386875; doi:10.1534/g3.116.038406)

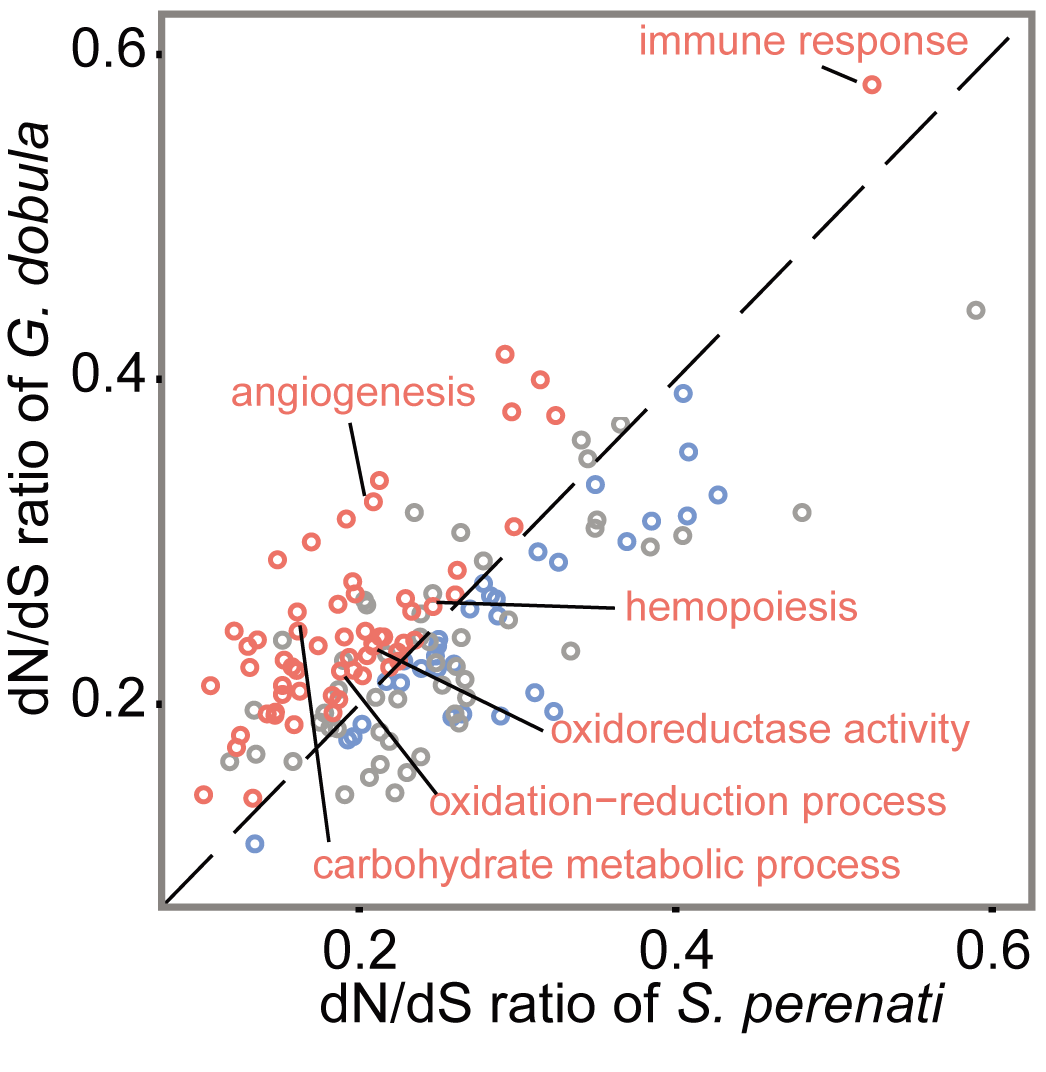

Supplement: Supplementary file 1 [file 1267FigureS1.tif]
